# Looking at the gender disparity in interventional radiology: a scoping review

**DOI:** 10.1093/bjr/tqae137

**Published:** 2024-08-05

**Authors:** Courtney Moffitt, Eloise Powell

**Affiliations:** Warwick Medical School, University of Warwick, Coventry, CV4 7AL, United Kingdom; Warwick Medical School, University of Warwick, Coventry, CV4 7AL, United Kingdom

**Keywords:** Gender Disparity, intervention, interventional radiology

## Abstract

**Objectives:**

The underrepresentation of women within interventional radiology (IR) is profound. This scoping review aims to evaluate the current literature on gender disparity within IR. To uncover relevant themes and research gaps to inform future research and to recommend changes aimed at increasing application and retention of women in IR

**Methods:**

A review of MEDLINE, EMBASE, and Web of Science was conducted. Specific inclusion and exclusion criteria were used to gather all relevant literature. Thematic analysis of included literature highlighted themes and commonalities between papers.

**Results:**

Of 396 articles, only 15 met the inclusion criteria. Many papers were excluded due to their lack of relevance to the topic. Thematic analysis identified 6 themes radiation exposure, mentorship, male dominance, work–life balance, research, and early exposure to IR.

**Conclusions:**

Recommendations relating to each theme have been made. Establishing a high-quality mentoring scheme, for medical students, is the priority. Followed by accurate information, regarding radiation safety and teaching opportunities provided by medical schools and placement trusts, to demonstrate the value of IR and the need for a representative workforce.

**Advances in knowledge:**

With little research based primarily within the United Kingdom, this review has amalgamated results from papers published internationally to highlight potential factors influencing the gender disparity within IR. Realistic recommendations and future points of research aimed at creating gender parity that are appropriate towards both the United Kingdom and global institutions have been suggested.

## Background

Despite a growing number of women entering medicine within the United Kingdom,^1^ a gender disparity is still evident within specialities.^2^ Female interventionalists remain underrepresented throughout the National Health Service (NHS). Of the 685 practicing interventionalists, only 12% of interventional radiologists working in the United Kingdom were female.^3^ The gender difference within interventional radiology (IR) has been further confirmed when looking at Society of Interventional Radiology memberships, with only 9.2% of active memberships held by females.^4^ Due to the advances in the technology, IR procedures have become more common and increasingly complex.^3^ Due to the increase in need, an expanding workforce is required to match the demand.

Guidelines set out by The Royal College of Radiologists state that timely access to IR is paramount to ensure patient safety, irrespective of hospital size.^3^ However, this guidance becomes harder to achieve, when accounting for the interventional radiologist shortfall of 28%, meaning there is a potential risk to service-user safety.^4^ This shortfall is further shown by 47 health boards not providing a 24/7 interventional service and 16% of health boards providing a 24/7 interventional service, on fewer than the recommended 6 consultants.^5^

Closing the gap will not only increase the female workforce but allow a larger overall workforce to cope with the demand faced within IR. Reducing the gender disparity is also important to the efficiency and effectiveness of the NHS, as it has been shown within a variety of industries that an improvement in critical analysis and decision-making took place with a mixed-gender group, rather than an all-male or female group.^6^

The justification and purpose of this scoping review is to look at the gender disparity that has occurred within IR, to identify contributing factors to the disparity and establish recommendations to how this can be rectified.

## Methods

Data have been collected from online databases available within the public domain, Embase (1964 to 2022 Week 41), Ovid Medline ^®^ ALL (1964 to October 20, 2022). Search terms identified for title search included “interventional Radiology,” “IR,” “interventionalist,” with the Boolean operator “and” combined with “Gender,” “Gap,” “Disparity,” “Difference,” Differences,” “Gender disparity,” “Female,” “Male” (see Supplementary Appendix S1 for the full search strategy).

Inclusion criteria covered studies that were peer-reviewed published articles, retrospective and prospective studies, commentaries, scoping reviews and those written in the English language. Participants included consultant or trainee interventional radiologist, qualified doctors yet to specialize into IR and medical students. Exclusion criteria included studies awaiting publication, conference abstracts, case control studies, case reports and case series, Letter to Editors, abstracts and studies written in a non-English language. All articles identified were reviewed by two independent reviewers (C.M. and E.P.) to determine if each paper met the inclusion criteria. The articles were quality assessed using Appraisal tool for Cross-Sectional Studies (AXIS)^7^ by the main author (see Supplementary Appendix S2 for a template). The AXIS quality assessment checklist was used to assess 11 of 15 papers, excluding those that are retrospective, commentaries or scoping reviews.

**Figure 1.**
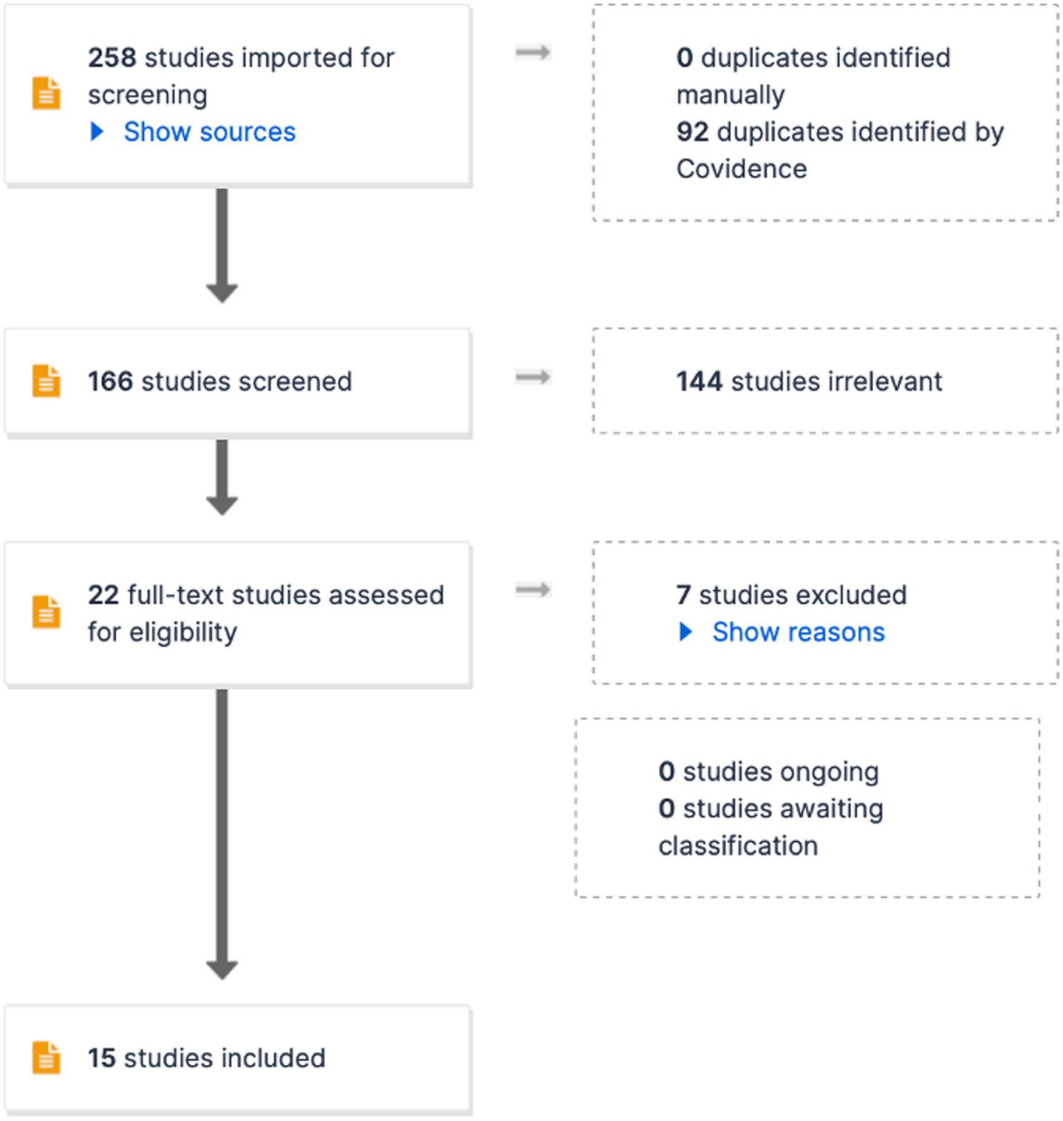
PRISMA 2020 flow diagram.

Of the 396 papers identified, 160 duplicate papers were removed. Two hundred and thirty-four papers were then screened by title and abstract where 207 studies were deemed irrelevant to the purpose of the scoping review. Twenty-seven studies then underwent a full-text screen, 13 papers were excluded, 6 due to being abstracts, 2 for being duplicated, 2 were deemed unrelated, 2 due to being a letter to editor and 1 due to being unable to obtain full text. Fifteen papers were deemed appropriate to be used in this scoping review. Each paper was assessed for suitability by 2 independent reviewers, when disagreement concerning eligibility occurred, each paper was discussed, and a final decision was made. (see Figure 1 for PRISMA 2020 flow diagram).^8^

A data extraction template was created using Microsoft Excel, a summary of the data extraction table is shown in Supplementary Appendix S3. Data were extracted by the main author and underwent thematic analysis identifying the main themes surrounding gender disparity in IR.

## Results

The 6 themes identified by thematic analysis include radiation exposure, mentorship, male dominance, work–life balance, research, and early exposure to IR. With 4 studies found to include research, 6 to include work–life balance, 9 to include mentorship, 6 to include male dominance, 4 to include early exposure and 4 to include radiation exposure as key themes.

### Radiation exposure

The perceived risk of radiation was deemed a deterrent for female doctors entering the speciality^9^^,^^10^ identified a particular concern regarding radiation exposure during pregnancy, making it a larger deterrent to females entering the speciality when compared to males. With correct radiation protection equipment research has expressed little concern regarding foetal health.^11^ With improvements in technology and radiation protection equipment, it has been shown that radiation exposure in IR is similar to natural background radiation.^12^ Despite this evidence, radiation safety was deemed a major concern for both male and female students when considering IR as a career.^13^

### Mentorship

Mentorship has been shown to be an important factor when recruiting those into the speciality.^14^ With mentorship being deemed an important component to a successful career within IR by both men and women, due to the guidance gained from a mentor being deemed varied and invaluable, from career advice to research guidance.^15^ However, women have reported less frequent access and lower levels of guidance from their mentor than their male counterparts, 29% compared to 64%, respectively.^15^ Further research has shown women value same-sex mentors highly when compared to men.^16^ With only 9% of mentors being female,^9^ the lack of female mentors have become a deterring factor to many female applicants.^17^

### Male dominance

Male dominance within the speciality has been shown to be a cause of concern for female medical students.^16^ Male dominance was also stated as a reason why women were at a disadvantage when pursuing a career within IR, as women felt that they were treated differently from their male counterparts by their superiors. Reasons varied from being perceived as less capable than male interventional radiologists to their being lower expectations of female interventionalists.^9^ The gender difference within IR has been further confirmed when looking at Society of Interventional Radiology memberships, with only 9.2% of active memberships held by females.^18^ Hence, male dominance has been stated as influencing gender disparity with IR.^9^

### Work–life balance

Work–life balance has been deemed an important factor to consider before specializing by both men and women, with 67% of female IR consultants agreeing that balancing work and family life to be challenging.^10^ Work–life balance has been stated as a reason why IR has not been deemed an attractive career for women to specialize in and why women believe they are at a disadvantage when pursuing a career in IR. However, this view has not been replicated within medical students, as both male and female participants equally found the work–life balance of the speciality a deterrent.^16^ This view was also expressed by Theodoulou et al^14^ finding that 48.2% of female respondents agreeing that they are able to maintain a positive work–life balance compared to only 45.2% of male respondents.

### Research

It has been shown men are more likely than women to believe to advance in IR it is important to conduct research.^15^ This is reinforced by men being more likely to agree than women that their institute encourages and values their engagement in research.^13^ This highlights a potential explanation of why there has not been a significant increase in female authorship within IR.^19^ When looking at publication numbers, at the level of professor, on average women achieve 17 publications fewer than their male counterparts, with only 8% of females reaching the level of professor compared to 15% of males.^18^ With female interventionalists receiving fewer industry payments in research when compared to their male counterparts.^20^ The lack of visible female representation has been shown a deterrent to other females entering the profession.^9^

### Early exposure to IR

It has been shown that early exposure to IR either through rotations, research opportunities or mentorship to be an important factor when aiming to recruit female applicants for the speciality.^15^^,^^21^ Early exposure, especially during medical school, allows questions to be answered and interests in the speciality to form.^16^ Personal interests have been shown the be a key factor when deciding on specialities.^13^ Further highlighting the importance of early exposure to IR and how this can reduce gender differences. It is also important to note that early exposure to IR has been deemed equally important to both male and female medical students, further highlighting the overall benefit, in terms of workforce size, to the profession that early access and understanding of the profession can have.^14^

## Discussion

Multiple themes in the scoping review have been identified in previous research; however, to the authors knowledge not all 6 themes together have been identified in a single other paper. Due to the scoping review inclusion criteria, doctors of all levels of specialism and medical students have been included. Very few papers have included this range of participants in a single study, showing how the influence of gender disparity on others joining the profession can impact a large population.

With increasing numbers of females entering the medical profession, specialities must recognize the demographic of future doctors is changing. With 64% of the 2021/2022 medical student cohort and 50% of those joining the workforce in 2021 being female.^1^ The need for specialities that are traditionally male dominated to recruit more female doctors has never been more critical. With only 12% of IR consultants being female, it shows that recruitment campaigns must be adapted to attract more females to the profession.^3^ Similar findings of gender disparity have been identified in other surgical specialties, namely Trauma and Orthopaedics where female representation is 7.3%. In contrast, female representation within ophthalmology is 49.7%,^2^ showing that gender parity can be achieved within a surgical based specialty. It is stipulated that the difference between gender disparity within different surgical specialities is a consequence of discrimination and gender-specific discouragement.^2^

Rectifying the gender difference within IR is important for multiple reasons. From a service-user perspective, improvements in patient satisfaction increase when being treated by a doctor that was similar to themselves, as this allowed trust to be built easier.^10^ Increasing gender diversity has also been shown to increase critical analysis and decision-making.^6^ As well as being better able to retain staff demonstrating the benefits of minimizing the gender gap, to not only the service users but to the department.^9^

The importance of accurate available data is paramount to decisions made about future careers. For instance, radiation safety has been shown the be a major deterrent for many females entering IR when compared to their male counterparts, this statement has been prevalent throughout available literature.^9^^,^^10^^,^^12^ The guidance surrounding radiation safety, particularly during pregnancy, states that no tissue reactions are expected to occur in a foetus when the dosage is below 100 mGy. The average dosage to IR radiologists is 0.35 mGy, showing the likelihood of exposure over 100 mGy to be low.^22^ Even with this guidance available, radiation exposure during pregnancy has been shown to play a major deterrent to females entering the profession.^10^^,^^23^ It is not clear from the papers included within this review if the guidance was known or if knowledge about radiation safety was due to misleading information.

The importance of mentoring in providing career guidance has been echoed throughout all specialities. It has been shown that those who receive mentoring are more likely to have better training outcomes aiding career progression with self-reported higher confidence levels.^24^ This reinforces the importance of mentorship within healthcare, highlighting the detrimental impact that lacking a mentor can have, further demonstrating how concerning it is that women are less likely to be able to access guidance from mentors than their male counterparts.^15^

Male dominance within the speciality is also a deterrent to female applicants.^16^ As there are very few females within the speciality, it means there are even fewer female mentors. By encouraging female colleagues to take up leadership roles, such as, mentorship, the visibility of females within IR will increase, this can only be beneficial to decreasing the gender gap with IR.

The ability to maintain a work–life balance has proved to be controversial, with many papers stating the inability to maintain a work–life balance as a deterrent to females entering the speciality.^9^^,^^10^ However, concerns about the time-intensive nature of the speciality have been echoed by both male and female medical students with Theodoulou et al^14^ finding that women reported more positively that a work–life balance was maintainable within IR, when compared to men, showing that work–life balance may not be as much of a deterrent as previously thought.

### Discusses strength and limitations of methods

Unfortunately, none of the articles in the scoping review included studies based solely in the United Kingdom. This means generalizing studies that are based outside of the United Kingdom to explain the gender disparity within the NHS is difficult.

Many of the studies required their participants to fill out questionnaires that included a mixture of open and closed questions and Likert scales. By using surveys as the primary method of data collection, it opens the results up to response bias, for instance, “In Medical school, I have had sufficient teaching of IR” agree or disagree.^10^ This question can be interpreted differently causing the results to be skewed. If interviews were incorporated, questions regarding interpretation of the question can be answered, giving a more representative answer. DeJonckheere and Vaughn^25^ stated in-depth interviews facilitate participants to express their thoughts, which will allow for more in-depth analysis to develop subthemes from them shedding further light on the disparity. This would be especially helpful to uncover if there are other reasons why the gender disparity in IR has occurred. The scale and size of the participant groups included within the scoping review have highlighted trends which are less likely to be due to chance.

A further limitation of this scoping review was that the literature was obtained from only 3 databases. This leaves the potential for relevant articles to have been missed if they were listed on databases other than Medline, Embase, or Web of Science. Data were extracted by the primary author, this meant time constraints were imposed on the data review and extraction process, and as a result information may have been missed.

However, the authors believe a strength of this research to be the inclusion of a broad range of participants ranging from medical students to interventionalists allowing for a range of perspectives to be given. This will allow a holistic approach to be taken when looking at the reasoning for gender disparity within IR and aid comprehensive recommendations aimed at all levels of training within IR. Unfortunately, the authors did not use a pre-published protocol.

## Recommendations

The papers utilized within this scoping review highlight the lack of literature surrounding gender disparity within IR that is based solely on UK data. It would be unreasonable to assume that all of the themes identified on the global platform are likely to apply to the United Kingdom and so recommendations made here can be integrated with this in mind. Due to this, key findings that apply to the NHS may be going undetected. The gender disparity in IR can only be corrected when the reasons for the gender disparity have been uncovered, hence why further research based in the United Kingdom is imperative.

Identifying the causal factors for the gender disparity can be uncovered by using a qualitative research method, either by conducting in-depth interviews or using focus groups including males and females at each level of training within IR. By using a qualitative research method, participants will be able to express individual views, aiding to the discussion surrounding gender disparity within IR.^26^ With these results, seminars aimed at medical school or newly qualified doctors can correct misleading information and ensure accurate information about the speciality is well known.

Medical schools also have a part to play in dispelling misinformation. This can be achieved by incorporating IR rotations/electives into the programme or inviting IR specialists to teach common IR procedures in the early years of the medical curriculum. Allowing medical students to be educated about the speciality and exposed to the profession at the start of their careers will not only make the speciality more visible to medical students but will also allow interest in the speciality to grow.

Male dominance is unfortunately the product of gender disparity. Encouraging female radiologists into leadership or mentoring roles will foster a more inclusive working environment, where male dominance is no longer a problem for those entering the profession.

Having a high-quality mentorship programme between students/trainees and interventional radiologists will mean many of the drawbacks to IR can be explained and misconceptions remediated. Mentorship can range from professional guidance to personal advice. This will mean questions surrounding a work–life balance can be answered and advice given. Advice surrounding research and how to get involved can also be gained from an effective mentoring programme, showing how mentorship can aid career progression. This could be achieved by the Royal College of Radiologists developing a mentoring programme aimed at medical students and junior doctors across the United Kingdom.

## Conclusion

This review has highlighted that more comprehensive research is needed to analyse the reasons for and the implications of the gender disparity within IR in the NHS. Overall, current research has uncovered multiple themes that contribute to the gender disparity on a global platform. It would be unreasonable to assume that all the themes identified on the global platform are likely to apply to the United Kingdom. Realistic recommendations to understand and decrease the gender disparity have been made to enhance the understanding of IR to medical students and qualified doctors to encourage future applications to the speciality.

## Supplementary Material

tqae137_Supplementary_Data
