# Supplementary material for: Looking at the gender disparity in interventional radiology: a scoping review
Source: Br J Radiol. 2024 Aug 5;97(1162):1622–6. doi: 10.1093/bjr/tqae137 (PMC11417366; doi:10.1093/bjr/tqae137)
Supplement: tqae137_Supplementary_Data [file tqae137_supplementary_data.zip › tqae137_Supplementary_Data/BJR 3 Appendices.docx]

**Appendices**

Appendix 1- Full search strategy


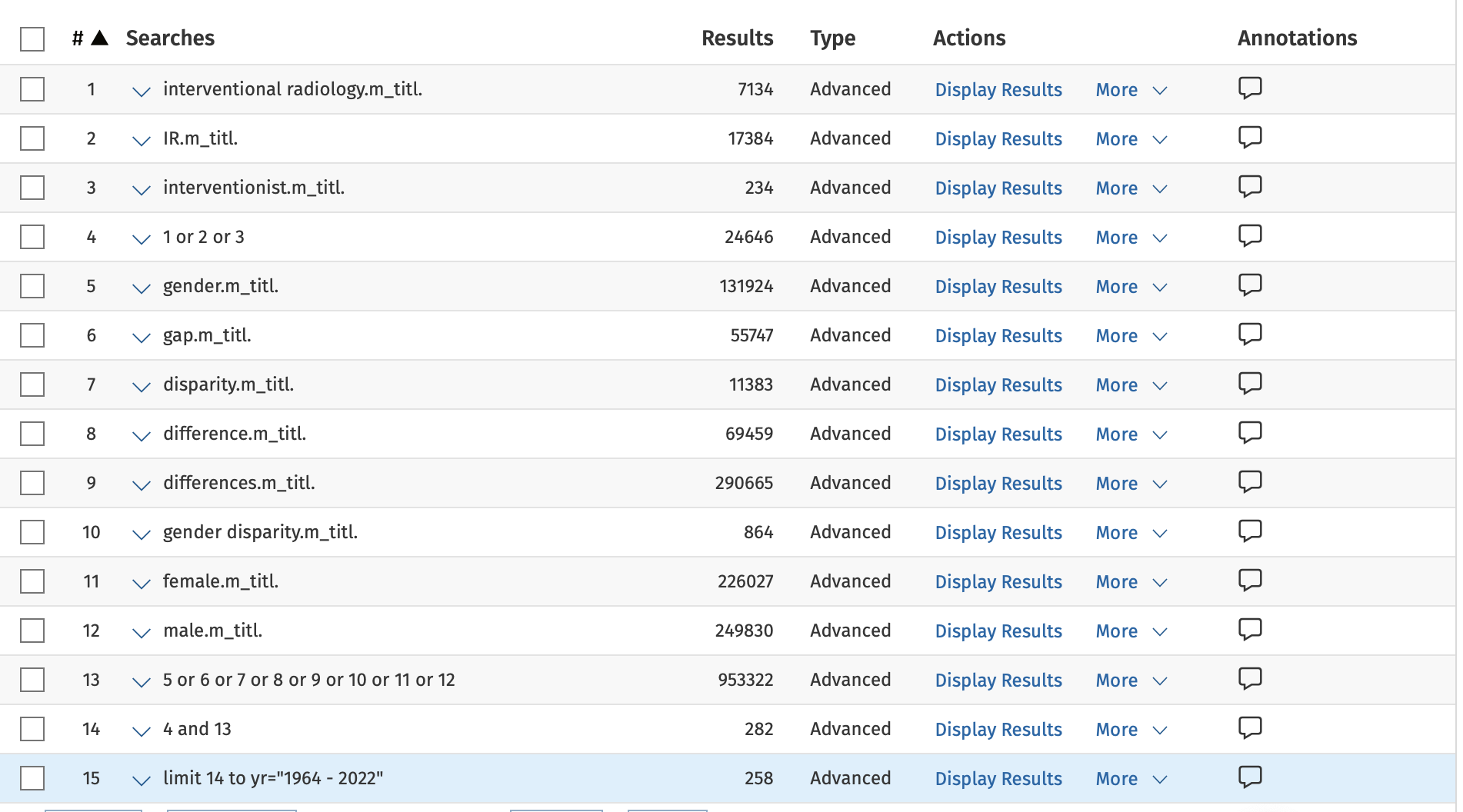

Appendix 2- Appraisal tool for Cross-Sectional Studies (AXIS) (Downes et al., 2016).

Appendix 3- Summary of Data Table

| **Author (year)** | **Title** | **Country of sample** | **Level of training** | **Study design** | **Female: Male** | **Main Themes** | **Study Quality- AXIS score out of 20** |
| --- | --- | --- | --- | --- | --- | --- | --- |
| Bernard C., Pommier R., Vilgrain V. and Ronot M. (2020) | Gender gap in articles published  in European Radiology and CardioVascular and Interventional Radiology: evolution between 2002 and 2016 | Austria, Belgium, China, France, Germany, Greece, Italy, Japan, South Korea, Spain, Sweden, Switzerland, Netherlands, turkey, UK, USA | IR fellow | Cross-sectional- | N/A | Research | 14 |
| Deipolyi, A. R.,  Becker, A. S.,  Covey, A. M.,  Chimonas, S. C.,  Rosenkrantz, A. B.,  Forman, H. P. and  Copen, W. A. (2020) | Gender disparity in industry  relationships with academic interventional radiology physicians | USA | Academic interventional radiology physicians | Cross-sectional | 108:734 | Research | 15 |
| Foo, M., Maingard, J., Wang, M., Kok, H., Chandra, R., Jhamb, A., Chong, W., Lee, M., Brooks, M. and Asadi, H., (2020) | Women in interventional  radiology: insights into Australia's gender gap | Australia | Junior Doctors yet to specialise | Cross-sectional | 175:158 | Work-life balance  Mentorship  Male Dominance | 12 |
| Huasen, B. Suwathep, P. Khan, A. Connor, B.  and holden, A. (2021) | Female medical student impression of interventional radiology: What can we do to improve this? | UK, Germany, Poland, Spain and New Zealand | Medical Student | Cross-sectional | 100:0 | Male Dominance  Mentorship  Early exposure to IR | 15 |
| Jaschke, W.,  Bartal, G.,  Trianni, A. and  Belli, A. M. (2018) | Fighting the Gender Gap in Interventional Radiology: Facts and  Fiction Relating to Radiation | N/A | N/A | Commentary | N/A | Radiation Safety | 1 |
| Li, O.,  Ross, M. and  Wiseman, D. (2021) | Women in interventional radiology: Exploring the Gender  Disparity in Canada | Canada | N/A | Scoping Review | N/A | Early exposure to IR  Radiation Safety  Mentorship  Work-life balance | 4 |
| Li, S., Sun, V.H., Galla, N., Salazar, G., Lewis, T., Ahmed, M. and Daye D (2022) | Gender-based survey analyis of research and mentoring in interventional radiology | USA | Radiology resident, fellow and medical student | Cross-sectional | 31:74 | Mentorship  Research | 14 |
| Matsumoto, M. M., Schultz, O., Jiang, T. and Navuluri, R. (2020) | Recruitment in Surgery and Interventional Radiology: Factors in Female Trainees' Specialty Decisions | USA | Medical Student | Cross-sectional | 48:0 | Mentorship | 15 |
| Matsumoto, M. M.,  Tullius, T. G., Jr. and  Navuluri, R. (2019) | Gender-Specific Factors Influencing Medical Students' Career  Choice of IR | USA | Medical Student | Cross-sectional | 31:35 | Mentorship  Male Dominance  Early exposure to IR | 14 |
| Moriarty, H. K.,  Clements, W.,  Zia, A.,  Connor, B. and  Goh, G. S. (2022) | The gender imbalance in Interventional Radiology in Australia  and New Zealand | Australia  New Zealand | Interventional Radiology Consultant, | Cross-sectional | 13:64 | Mentorship  Work-Life balance  Radiation Exposure | 17 |
| Parikh, R.,  Shamimi-Noori, S.,  Gade, T.,  Nadolski, G. and  Hunt, S. (2021) | Demographic trends in female interventional radiology trainees with the advent of the integrated interventional radiology residency | USA | Radiology trainee | Cross-sectional | N/A | Work-life balance  Mentorship | 16 |
| Theodoulou, I.,  Dost, S.,  Burrows, V.,  Lyall, F.,  Wah, T. M. and  Makris, G. C. (2022) | The interventional radiology gender gap: perspectives from the  international IR training survey | Canada, USA, South America, UK  Europe, Middle East, Asia, Australia | Radiology trainee | Cross-sectional | N/A | Early exposure to IR  Mentorship  Work-life balance | 15 |
| Wah, T. M. and  Belli, A. M. (2018) | The Interventional Radiology (IR) Gender Gap: A Prospective Online Survey by the Cardiovascular and Interventional Radiological Society of Europe (CIRSE) | UK, Italy, Germany, Sweden, Spain, Netherlands, France, USA | N/A | Cross-sectional | 149:0 | Work-life balance  Male Dominance  Radiation exposure | 12 |
| Wang, J.,  Khurshid, K.,  Jalal, S.,  Nicolaou, S.,  White, S. B.,  Englander, M. J.,  Salazar, G. M. and  Khosa, F. (2019) | Influence of academic productivity on gender disparity in academic interventional radiology | Canada, USA | Assistant professor  Associate Professor  Professor | Retrospective review | N/A | Research  Male Dominance | 14 |
| Wang, M.,  Laguna, B.,  Koethe, Y.,  Lehrman, E.,  Kumar, V. and  Kohi, M. P. (2019) | Bridging the Gender Gap in the Society of IR: A Benchmark Study | N/A | Medical Student  Radiology Resident  Radiology fellow | Retrospective review | N/A | Male Dominance | 13 |
